# The Role of Natural Orifice Specimen Extraction in Modern Surgery: A Narrative Review

**DOI:** 10.7759/cureus.100162

**Published:** 2025-12-26

**Authors:** Mark Salib, John Salib, Murali K Manikkavelu, Timothy J Stear

**Affiliations:** 1 Medicine, St. George's University School of Medicine, St. George's, GRD; 2 General Surgery, Community First Medical Center, Chicago, USA; 3 Medicine, American University of Antigua, St. John, ATG; 4 General Surgery, Resurrection Medical Center, Chicago, USA

**Keywords:** laparoscopic surgery, minimally invasive surgery, natural orifice specimen extraction, nose, nose surgery, specimen extraction route, specimen retrieval, transgastric specimen extraction, transrectal specimen extraction, transvaginal specimen extraction

## Abstract

Natural Orifice Specimen Extraction (NOSE) is an innovative, minimally invasive technique that combines laparoscopic or robotic resection with specimen retrieval through natural orifices, eliminating the need for abdominal extraction incisions. By avoiding additional incisions, NOSE reduces postoperative pain, wound complications, and recovery time, while preserving oncologic integrity. It has been widely applied in colorectal and gynecologic procedures, demonstrating feasibility, safety, and improved patient-centered outcomes. This review synthesizes current evidence on surgical indications, technical considerations, postoperative recovery, complications, oncologic outcomes, and patient-reported metrics. Robotic-assisted NOSE is also discussed as a means to overcome anatomical challenges and expand procedural applicability. Despite promising results, gaps remain in long-term functional outcomes, standardized protocols, and economic analyses. Continued research is needed to refine techniques, broaden indications, and optimize patient outcomes. NOSE represents a transformative approach within minimally invasive surgery, potentially improving recovery, satisfaction, and overall surgical quality.

## Introduction and background

Minimally invasive surgery has transformed modern surgical practice by reducing patient morbidity, enhancing recovery, and improving cosmetic outcomes compared to traditional open approaches [1]. Laparoscopic and robotic techniques have become standard in many surgical specialties, particularly colorectal surgery, where they have demonstrated advantages in postoperative pain reduction, length of hospital stay, and wound-related complications [2]. However, even in conventional minimally invasive procedures, specimen extraction typically requires an abdominal incision. This additional incision, often performed through an extended trocar site or a small laparotomy, has been associated with pain, wound infection, hernia formation, and suboptimal cosmetic outcomes [3]. Notably, Pfannenstiel incisions, a low transverse suprapubic approach, are associated with a lower risk of incisional hernia compared with small midline laparotomies, making them a preferred extraction site when feasible [2-4].

To overcome these limitations, surgeons have explored strategies to avoid abdominal incisions altogether. Natural orifice specimen extraction (NOSE) has emerged as an innovative technique that eliminates the need for an abdominal extraction site by removing surgical specimens through natural orifices, most commonly the anus or the vagina in colorectal practice [4]. In the past, transgastric approaches performed via endoscopy have been described, but these utilize a natural orifice transluminal endoscopic surgery (NOTES) approach and do not involve laparoscopic resection. Early NOTES efforts in the 2000s sparked interest in using natural access points for intra-abdominal procedures [5]. Although NOTES faced challenges in instrumentation and safety, avoiding abdominal incisions influenced the development of NOSE, which has gained broader clinical acceptance. Unlike NOTES, NOSE leverages established laparoscopic resection techniques, introducing innovation only at the stage of specimen retrieval, thereby lowering the barrier to adoption [6]. By combining laparoscopic resection with natural orifice extraction, NOSE represents a hybrid technique that retains the advantages of minimally invasive surgery while further minimizing abdominal wall trauma.

Since its introduction, NOSE has been increasingly adopted in colorectal surgery, particularly for procedures such as anterior resections, sigmoid colectomies, and low anterior resections. Other specialties have also investigated the approach, including gynecology and upper gastrointestinal surgery [7]. Early reports suggested potential benefits in reducing postoperative pain and improving cosmetic outcomes, which stimulated interest in evaluating NOSE more rigorously in clinical trials and meta-analyses [8].

Current evidence on NOSE consists of a heterogeneous mix of randomized controlled trials (RCTs), propensity score-matched cohort studies, meta-analyses, and narrative reports. While several meta-analyses suggest improvements in postoperative pain, recovery, and incision-related morbidity, the literature varies in study design, patient selection, extraction route, and outcome reporting, leading to inconsistent conclusions and uncertainty regarding generalizability. Despite a growing body of randomized trials, meta-analyses, and observational studies, important knowledge gaps remain regarding the consistency of clinical benefit across patient populations, the magnitude of complication reduction, the oncologic safety of natural-orifice extraction in malignant disease, and the integration of NOSE into contemporary laparoscopic and robotic workflows.

Despite these promising findings, NOSE has not been universally adopted. Concerns remain regarding technical feasibility, learning curve, patient selection, and critically, oncologic safety, particularly in colorectal cancer resections, though similar concerns have been noted in exploratory applications to gynecologic and upper gastrointestinal surgery [9,10]. Additional considerations include the potential for iatrogenic anal sphincter injury during transanal specimen retrieval, as well as questions about contamination risk, tumor cell dissemination, and margin retrieval adequacy. Nevertheless, current evidence suggests that oncologic outcomes are not compromised [10,11]. 

This narrative review aims to synthesize and contextualize the current evidence on NOSE in minimally invasive surgery. Specifically, this review examines: (1) postoperative recovery and pain-related outcomes; (2) complication profiles, including incision-related and natural-orifice-specific events; (3) oncologic adequacy and long-term survival outcomes in colorectal cancer; and (4) patient-reported and cosmetic outcomes. In addition, the review discusses technical considerations, patient selection, the role of robotic-assisted NOSE, and future directions for research and clinical adoption. By integrating data across diverse study designs, this review seeks to clarify the clinical role of NOSE and provide practical guidance for surgeons and clinicians unfamiliar with the technique.

History and background 

The development of NOSE is closely linked to the evolution of minimally invasive surgery over the past few decades. The widespread adoption of laparoscopic techniques in the late 20th century established the foundation for approaches that minimize abdominal wall trauma [12]. Early successes in laparoscopic colectomy demonstrated that complex intra-abdominal procedures could be performed safely with smaller incisions, reduced postoperative pain, and faster recovery, which encouraged the exploration of even less invasive alternatives [12].

In the early 2000s, NOTES introduced the concept of accessing the abdominal cavity entirely through natural orifices, such as the stomach, rectum, or vagina, without any external incisions [13]. Although NOTES was conceptually transformative, its clinical adoption was limited by technical challenges, including closure of visceral entry points, instrument maneuverability, and infection control [13]. These limitations inspired the development of NOSE, which preserved standard laparoscopic resection techniques while innovating only in the specimen extraction phase. By narrowing the scope to extraction, NOSE offered a safer and more practical alternative that could be integrated into existing minimally invasive practices [11,13].

The first clinical applications of NOSE emerged in the 1990s, when pioneering surgeons reported transanal and transvaginal retrieval of colorectal specimens following laparoscopic resection [14]. These early experiences were limited to select patient populations with small tumors and favorable anatomy, but they demonstrated feasibility and encouraged further refinement of the technique [12,14]. Gradual improvements in laparoscopic instruments, specimen retrieval devices, and patient selection criteria allowed NOSE to be applied more broadly in colorectal procedures, particularly low anterior resections and sigmoid colectomies [14].

By the mid-2000s, larger case series evaluated outcomes systematically, focusing on reducing incision-related complications such as wound infection, hernia formation, and postoperative pain [15]. Initial concerns regarding intraperitoneal contamination, tumor cell dissemination, and technical difficulties in natural orifice closure prompted careful patient selection and procedural standardization [12,15].

The acceptance of NOSE in oncologic colorectal surgery marked a pivotal moment in its history. Studies demonstrated that specimens could be extracted through natural orifices without compromising oncologic principles, including negative resection margins and adequate lymph node harvest [16]. Multicenter experiences and meta-analyses later confirmed these findings, showing comparable long-term survival and recurrence rates to conventional laparoscopic surgery and highlighting the benefits in postoperative recovery and cosmetic outcomes [11,16].

Currently, NOSE is widely practiced in colorectal surgery, with transanal and transvaginal routes being the most common. Interest in expanding its application to gynecologic and upper gastrointestinal procedures continues, reflecting the adaptability of the technique [14,16]. Its evolution illustrates how incremental innovation, guided by clinical evidence, can transform surgical practice and improve patient outcomes.

## Review

Methods

**Figure 1:**
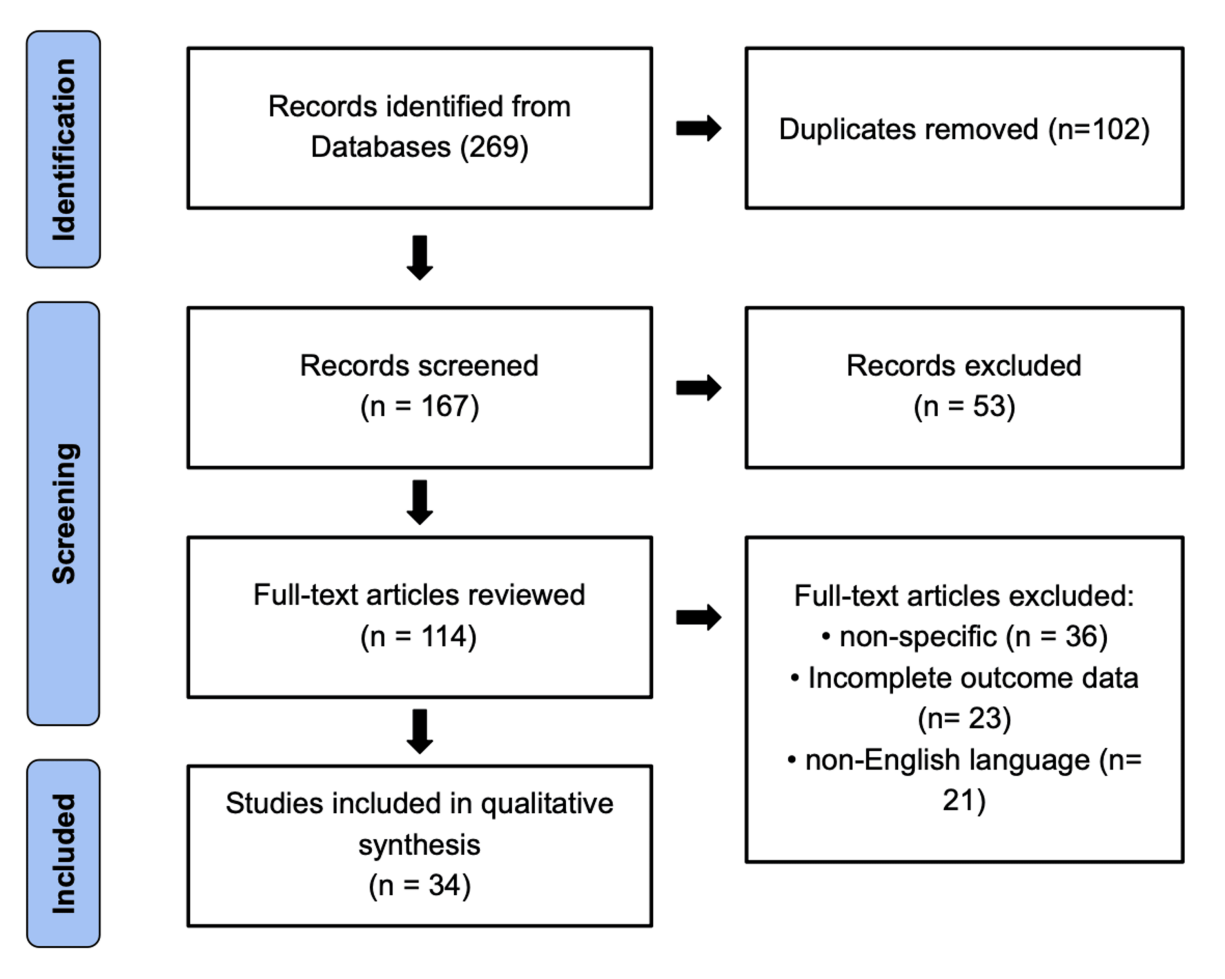
PRISMA flow diagram of study selection for the role of NOSE in modern surgery This flowchart illustrates the systematic identification, screening, eligibility, and inclusion process for studies evaluating the role of NOSE in modern surgery. A total of 269 records were identified through database searching, with 102 duplicates removed. After screening 167 records, 53 were excluded based on titles and abstracts. Of the 114 full-text articles reviewed, 80 were excluded for reasons including non-specific focus (n = 36), incomplete outcome data (n = 23),  and non-English language (n = 21). Ultimately, 34 studies were included in the qualitative synthesis. PRISMA: Preferred Reporting Items for Systematic Reviews and Meta-Analyses; NOSE: Natural orifice specimen extraction

This study is a qualitative narrative review designed to synthesize current evidence on the applications, outcomes, and safety profile of NOSE surgery (NOSES) across gastrointestinal and gynecologic procedures. The review was conducted in accordance with methodological principles adapted from the Preferred Reporting Items for Systematic Reviews and Meta-Analyses (PRISMA) guidelines to ensure transparency and reproducibility (as depicted in Figure 1) [17].

A comprehensive literature search was conducted across the PubMed, Scopus, Embase, and Google Scholar databases from January 2010 to October 2025. The search included a combination of Medical Subject Headings (MeSH) and free-text keywords: “Natural Orifice Specimen Extraction,” “NOSE,” “NOSES,” “Laparoscopic Colorectal Surgery,” “Transvaginal Specimen Extraction,” “Transanal Specimen Extraction,” “Minimally Invasive Surgery,” “Robotic Surgery,” and “Outcomes.” Reference lists of relevant articles were manually screened to identify additional eligible studies.

The inclusion criteria included studies that reported outcomes of NOSES or NOSE techniques in any gastrointestinal or gynecologic procedure, studies that were original clinical studies (RCTs, cohort studies, case-control studies, prospective/retrospective series, or case reports) or secondary evidence (systematic reviews, meta-analyses, or narrative reviews), studies that reported quantitative or qualitative outcome measures, including operative metrics, postoperative recovery, complications, oncologic outcomes, or patient-reported measures, and finally, studies that were published in English in peer-reviewed journals. The exclusion criteria include abstracts, editorials, commentaries, conference proceedings, or animal studies. Studies not describing specimen extraction via natural orifice routes (e.g., purely transabdominal or open approaches), as well as duplicate publications or overlapping data from the same cohort.

Four independent reviewers screened all retrieved titles and abstracts. Full texts of potentially eligible articles were then reviewed for inclusion. The reviewers independently extracted relevant data on sample size (N), indication, intervention (dose/route), comparator, outcome measures, baseline and follow-up values, follow-up duration, and key findings. Any discrepancies were resolved through consensus-based discussion. To minimize bias, each study was assessed for methodological quality using design-appropriate tools: the Cochrane Risk of Bias Tool for randomized trials, the Newcastle-Ottawa Scale for observational studies, and the Scale for the Assessment of Narrative Review Articles (SANRA) criteria for narrative reviews. Inter-reviewer agreement was quantified using Cohen’s κ coefficient. The extracted data were tabulated and summarized in Table 1. 

**Table 1:** Summary of included studies evaluating NOSES This table summarizes key findings from narrative reviews, systematic reviews, meta-analyses, clinical studies, and case reports evaluating NOSE across gastrointestinal and gynecologic procedures. NOSE: Natural orifice specimen extraction; NOSES: Natural orifice specimen extraction surgery; SSI: Surgical site infection; LOS: Length of stay; VAS: Visual analog scale; OS: Overall survival; DFS: Disease-free survival; PD: Pancreaticoduodenectomy; PRO: Patient-reported outcome; PSM: Preventive and social medicine; QoL: Quality of life; RCT: Randomized controlled trial; ssNOSES: single-stapling natural orifice specimen extraction surgery

First Author (Year)	Study Type	Population/Indication	Intervention	Comparator	Outcome Measures	Results (Baseline → Follow-up)	Key Findings
Zhang (2022) [1]	Narrative Review	Minimally invasive colorectal surgery	NOSE	Conventional laparoscopy	Operative time, pain, cosmesis, recovery	↓ Pain, ↓ LOS, improved cosmesis	Positions NOSE as the next step in minimally invasive colorectal surgery
Brincat (2022) [2]	Meta-analysis	Colorectal cancer	NOSE	Transabdominal extraction	LOS, pain, SSI, conversion	↓ LOS (−1.2d), ↓ SSI, ↓ pain (VAS −0.9)	NOSE reduces pain and wound complications without affecting oncologic outcomes
Izquierdo (2018) [3]	Narrative Review	Colorectal disease	NOSE	—	Patient selection, outcomes	—	Defines selection criteria (BMI <30, tumor <5 cm); emphasizes feasibility
Chin (2020) [4]	Meta-analysis	Colorectal cancer	NOSE	Conventional laparoscopy	Operative time, pain, LOS, recurrence	↓ LOS (−1.4d), ↓ pain, similar recurrence	Confirms perioperative benefits with equivalent oncologic outcomes
Salim (2023) [5]	Single-center Prospective	Robotic sigmoidectomy	Robotic NOSE	Robotic conventional	Operative time, recovery, complications	↓ Pain, 0 wound infections	Safe and feasible robotic NOSE approach
Kar (2024) [6]	Systematic Review & Meta-analysis	Bowel resection for endometriosis	NOSE	Mini-laparotomy	LOS, pain, cosmesis, complications	↓ LOS, ↓ pain (VAS −1.3), improved cosmesis	Promising alternative to minilaparotomy for endometriosis bowel resection
Thakkar (2021) [7]	Narrative Review	Colorectal cancer	NOSE	Conventional lap	Feasibility, outcomes	—	Highlights hybrid benefits of laparoscopy and transnatural access
Mui (2025) [8]	Systematic Review	Diverticular disease	Laparoscopic NOSE	Conventional laparoscopy	LOS, recovery, SSI	↓ LOS (−0.9d), ↓ SSI	NOSE is safe and effective for benign diverticular resections
Zhou (2022) [9]	Meta-analysis (RCTs)	Colorectal cancer	Laparoscopic NOSE	Conventional surgery	OS, DFS, complications	Similar OS/DFS; ↓ wound complications	Maintains oncologic equivalence with fewer wound issues
Wang (2022) [10]	Meta-analysis	Colorectal cancer	NOSE	Conventional lap	Operative, oncologic, QoL	↓ Pain, ↓ SSI, no OS/DFS difference	Supports NOSE safety with comparable long-term results
Zhang (2020) [11]	Case Series	PD	Laparoscopic PD + NOSE	Historical PD	Feasibility, complications	Feasible in select cases	Demonstrates feasibility of NOSE for PD in expert settings
Wang (2022) [12]	Systematic Review & Meta-analysis	Right hemicolectomy	NOSE	Conventional lap	Safety, oncologic outcomes	Comparable safety; ↓ pain, ↓ LOS	Safe for right hemicolectomy, equal oncologic outcomes
Zhang (2022) [13]	Narrative Review	Gastric cancer	NOSE gastrectomy	—	Feasibility, selection	—	Highlights developmental potential in gastric surgery
Yu (2023) [14]	Comparative Cohort	Right hemicolectomy	NOSE	Small-incision lap	Operative metrics, recovery	Improved cosmesis & recovery	NOSE yields faster recovery with equivalent oncologic safety
Shen (2020) [15]	Technical Report	Left colon cancer	ssNOSES	Conventional	Feasibility, anastomotic integrity	Successful cases reported	Demonstrates single-stapling NOSES feasibility
Gundogan (2023) [16]	Retrospective Series	Laparoscopic colorectal resections	NOSE	—	Feasibility, complications	Acceptable complication rates	Large single-center validation of NOSE feasibility
Page (2021) [17]	Reporting Guideline/Methodological Framework	—	—	—	—	—	PRISMA 2020 provides updated, standardized recommendations and flow diagram requirements for transparent reporting of systematic reviews.
Pompeu (2025) [18]	Systematic Review & Meta-analysis (PSM)	Right colon cancer	NOSE	Conventional lap	Safety, oncologic outcomes	Comparable outcomes; periop benefits	Confirms safety in matched analyses
Wolthuis (2015) [19]	RCT	Laparoscopic colectomy	With vs without NOSE	Conventional	Pain, recovery, complications	Improved early pain scores	One of first RCTs validating NOSE safety
Ma (2015) [20]	Meta-analysis	Colorectal disease	NOSE	Conventional lap	Short-term outcomes	↓ Pain, ↓ LOS	Demonstrated periop benefits without oncologic harm
Wolthuis (2015) [21]	Prospective PRO Study	Post-lap colectomy	NOSE	Conventional	Cosmesis, satisfaction	High cosmesis satisfaction	Excellent patient-reported cosmesis after NOSE
Cao (2025) [22]	Meta-analysis	Colorectal cancer	NOSE	Conventional	Short- & long-term efficacy	↓ LOS, ↓ pain, equal oncologic	Confirms periop benefits, neutral oncologic impact
Zengin (2022) [23]	Narrative Review	Colorectal cancer	NOSE	—	Complications, management	—	Outlines complication profiles & management strategies
Costantino (2011) [24]	Prospective Comparative	Left colorectal resections	Transanal vs transabdominal	Transabdominal	Peritoneal contamination	No significant contamination difference	No evidence of bacterial safety compromise
Zhang (2022) [25]	Comparative Cohort	Rectal cancer	NOSES	Non-NOSES	Short/long-term outcomes	Improved recovery; similar oncologic	Validates safety in rectal NOSES
Ouyang (2020) [26]	Comparative	Colorectal cancer	NOSES	Conventional lap	Bacteriology, oncologic	No difference in oncologic outcomes	Addresses contamination fears; safe in trials
Tang (2021) [27]	PSM Study	Colorectal cancer	NOSES	Conventional lap	Periop, complications, oncologic	↓ Pain, ↓ LOS, similar oncologic	Supports NOSES periop advantage
Zhou (2019) [28]	Comparative Cohort	Anterior resection	Transanal NOSES	Mini-lap	Recovery, survival	Improved early recovery, equal survival	Early recovery benefit maintained long term
Gao (2020) [29]	Retrospective Mono-institutional	Female robotic anterior resection	Transvaginal NOSE	Mini-lap	Short-term, oncologic	Better cosmesis, equal oncologic	Safe in selected female pts
Lee (2017) [30]	Meta-analysis	Extraction site hernias	NOSE	Mini-lap	Hernia incidence	↓ Hernia incidence	Avoiding incision reduces hernia risk
Fang (2025) [31]	Case Series	Rectal cancer	ssNOSES	Conventional	Anastomotic outcomes	Safe early results	Early safety confirmed for ssNOSES
Liu (2025) [32]	Meta-analysis	Colorectal cancer	NOSE	Conventional	CRP, WCC, complications	↓ Inflammatory markers postop	Suggests reduced surgical stress
Sarofim (2025) [33]	Review	Diverticular disease	NOSE	Conventional	Technique, inflammatory markers	Improved outcomes, lower markers	Favorable NOSE results for diverticular disease
Huang (2025) [34]	Narrative Review	Colorectal cancer	NOSE	—	Evidence, development	—	Summarizes evidence, calls for standardization
Fang (2025) [35]	Retrospective Cohort	Stage II–III rectal cancer	NOSE	Traditional lap	Long-term oncologic	Improved DFS, OS (reported)	Reports superior long-term outcomes; needs validation

Given the heterogeneity in study design, patient populations, and outcome measures, a qualitative narrative synthesis was performed rather than a quantitative meta-analysis. Emphasis was placed on the consistency of clinical outcomes across different NOSES techniques, patient selection criteria, perioperative safety, and oncologic equivalence compared to conventional laparoscopic or open approaches.

Surgical indications and techniques 

NOSE is primarily employed in colorectal surgery, where it complements laparoscopic resections for conditions such as rectal cancer, sigmoid colon cancer, diverticulitis, and inflammatory bowel disease [2,10]. The procedure is particularly advantageous for low anterior resections and sigmoid colectomies, as these allow direct transanal or transvaginal access for specimen extraction [2,10,16]. Its application has also been explored in gynecologic procedures, including hysterectomy and adnexal mass removal, where transvaginal extraction provides a minimally invasive alternative to traditional laparoscopic specimen retrieval [16].

Patient selection is a critical determinant of procedural success. Ideal candidates typically have small to moderate-sized specimens, favorable anatomy, and no significant history of pelvic inflammatory disease or prior pelvic surgery that could complicate natural orifice access [18]. Tumor characteristics, such as location and stage, are also considered, as very large or bulky specimens may not be amenable to transanal or transvaginal extraction without risking tissue fragmentation or contamination [16]. In addition, surgeons consider patient comorbidities, body habitus, overall surgical risk, and patient gender when assessing a patient’s suitability for NOSE. Patient gender is particularly relevant because transvaginal extraction is only feasible in female patients and may influence route selection.

Technically, NOSE integrates standard laparoscopic resection with natural orifice extraction. After mobilization and resection of the target tissue, the specimen is placed into a protective retrieval bag and guided through the chosen natural orifice. Transanal extraction is most commonly used in colorectal procedures, particularly for rectal and sigmoid resections. In contrast, transvaginal extraction is utilized in female patients for specimens that are difficult to extract transanally [12,14]. As depicted in Table 2, the route choice depends on the specimen size, patient anatomy, and surgeon experience.

**Table 2:** Common procedures, extraction routes, and evidence-based considerations for NOSE Summary of common surgical procedures utilizing NOSE, including the most frequently used extraction routes and evidence-based considerations. Key considerations highlight the clinical advantages, including safety, oncologic integrity, postoperative recovery, and cosmetic outcomes, with supporting references [12,14,16,18]. NOSE: Natural orifice specimen extraction

Procedure	Most Common Extraction Route	Key Considerations
Low anterior resection	Transanal	Enables safe extraction for tumors ≤6 cm with minimal postoperative pain; preserves oncologic integrity with adequate distal margins and lymph node retrieval [12,14]
Sigmoid colectomy	Transanal	Reduces incision-related complications and accelerates recovery; specimen handling in a retrieval bag prevents tumor fragmentation and maintains sterility [12,14]
Rectal cancer	Transanal	Demonstrated oncologic equivalence to conventional laparoscopy, with comparable recurrence rates and improved cosmetic outcomes [12,16]
Hysterectomy/adnexal masses	Transvaginal	Minimizes abdominal wall trauma, lowers postoperative pain, and maintains tissue integrity; associated with rapid return to daily activities [16]
Other selected colorectal resections	Transanal or transvaginal	Feasible for moderate-sized specimens when patient anatomy permits; optimizes recovery and reduces wound complications without compromising oncologic safety [12,18]

Closure of the natural orifice site is essential to minimize the risk of contamination and infection. In transanal extraction, closure may involve hand-sewn sutures or stapling devices, while transvaginal extraction typically requires layered closure to restore normal anatomy [18]. Surgeons also employ meticulous irrigation and prophylactic antibiotics for perioperative infection control.

The evolution of instruments and retrieval devices has facilitated the broader adoption of NOSE. Specialized specimen bags, flexible laparoscopic instruments, and articulating staplers have enabled safe and efficient extraction, reducing operative time and minimizing tissue trauma. The technique maintains the oncologic principles of resection while enhancing patient-centered outcomes, including reduced pain, quicker recovery, and improved cosmesis [12,16].

In summary, NOSE is indicated for a subset of patients undergoing colorectal and gynecologic surgery where natural orifice extraction is feasible. Careful patient selection, appropriate procedural planning, and meticulous surgical technique are critical for optimizing outcomes and minimizing complications [12,18].

Clinical outcomes

Postoperative Recovery

Evidence consistently demonstrates that NOSE improves short-term recovery parameters compared to conventional laparoscopic surgery. In a randomized clinical trial, patients undergoing NOSE had significantly lower postoperative pain scores (mean maximum visual analog scale (VAS) 2.1 vs 3.5; P < 0.001) and required less analgesia, including lower patient-controlled epidural use (116 mL vs 221 mL; P < 0.001) and markedly fewer needing morphine analogues (1/20 vs 10/20; P = 0.003) compared with conventional laparoscopic colectomy [18]. A meta-analysis of nine studies involving 837 patients confirmed these findings, showing lower pain scores (weighted mean difference (WMD) -1.43, 95% CI -1.95 to -0.90), earlier return of bowel function (WMD -0.59 days, 95% confidence interval (CI) -0.78 to -0.41), and shorter hospital stay (WMD -0.62 days, 95% CI -0.95 to -0.28) in NOSE compared with conventional laparoscopic colectomy [19]. These improvements in pain control and early recovery facilitate faster mobilization and postoperative rehabilitation.

Another well-documented benefit is the earlier return of gastrointestinal function. Meta-analyses have shown that patients resume flatus and bowel movements significantly sooner after NOSE compared with conventional laparoscopic colectomy (WMD -0.59 days, 95% CI -0.78 to -0.41) [19,20]. This accelerated recovery is likely related to reduced abdominal wall trauma and is typically accompanied by earlier progression to oral intake in enhanced recovery pathways.

Enhanced recovery protocols also appear to integrate well with NOSE. Patients typically mobilize earlier postoperatively, which facilitates earlier participation in physiotherapy and discharge planning. Meta-analyses confirm that NOSE is associated with a shorter hospital stay, with a pooled reduction of approximately 0.6-0.8 days compared to conventional laparoscopic colectomy, without compromising safety outcomes [19,20].

Finally, NOSE offers a unique recovery advantage in cosmesis and patient-reported outcomes (PROs). The absence of a visible abdominal extraction scar improves patient satisfaction and self-image. In a matched cohort study, Wolthuis et al. found that patients who underwent NOSE colectomy had significantly better body image scores (median 15 vs 18; P = 0.027) and Patient Scar Assessment Questionnaire (PSAQ) scores (median 56 vs 71; P = 0.002) compared with those who underwent conventional laparoscopic colectomy [21]. These cosmetic and body image benefits translate into improved psychosocial well-being and overall quality-of-life perceptions, aligning with modern expectations for minimally invasive surgery.

As shown in Table 3, NOSE provides clear advantages in postoperative recovery compared with conventional laparoscopic colectomy. Randomized trials and meta-analyses consistently demonstrate significantly lower postoperative pain, reduced analgesic requirements, faster return of bowel function, and shorter hospital stays. Enhanced recovery is further supported by improved cosmetic satisfaction and body image scores due to the absence of an abdominal extraction incision. Collectively, these findings confirm that NOSE accelerates short-term functional recovery without compromising safety.

**Table 3:** Postoperative recovery outcomes after NOSE compared with conventional laparoscopic surgery Values are reported as means or proportions as extracted from RCTs and meta-analyses. Effect sizes are presented as WMD with 95% CIs where applicable. Postoperative pain was assessed using the VAS. Cosmetic outcomes were evaluated using validated instruments including the BIQ and PSAQ. Opioid consumption reflects epidural volume administered and proportion of patients requiring postoperative morphine. Data synthesized from Wolthuis et al., Ma et al., and Pompeu et al. [18–21]. BIQ: Body Image Questionnaire; CI: Confidence interval; NOSE: Natural orifice specimen extraction; PSAQ: Patient Scar Assessment Questionnaire; RCT: Randomized controlled trial; VAS: Visual analog scale; WMD: Weighted mean difference

Outcome	NOSE	Conventional Laparoscopy	Effect Size/Statistics
Postoperative pain (VAS)	Mean maximum VAS: 2.1	Mean maximum VAS: 3.5	WMD –1.43 (95% CI –1.95 to –0.90); P < 0.001
Opioid consumption	Epidural use: 116 mL; Morphine required: 1/20 patients	Epidural use: 221 mL; Morphine required: 10/20 patients	P = 0.003
Time to first flatus/bowel movement	Mean: 1.8 days	Mean: 2.4 days	WMD –0.59 days (95% CI –0.78 to –0.41)
Time to oral intake resumption	Mean: 1.2 days	Mean: 1.8 days	Mean difference –0.6 days
Length of hospital stay	Mean: 4.2 days	Mean: 4.8 days	WMD –0.62 days (95% CI –0.95 to –0.28)
Cosmetic satisfaction (BIQ/PSAQ scores)	BIQ score: lower (better); PSAQ score: higher	BIQ score: higher (worse); PSAQ score: lower	P < 0.01

Complications

One of the main advantages of NOSE is the reduction in incision-related complications, particularly surgical site infections (SSIs), wound dehiscence, and incisional hernias. By eliminating the need for an abdominal extraction incision, NOSE significantly lowers these risks. In a meta-analysis including 4,637 patients, NOSE was associated with significantly lower odds of wound infection (odds ratio (OR) 0.22, 95% CI 0.13-0.38) and incisional hernia (OR 0.24, 95% CI 0.11-0.54) compared with conventional laparoscopic colectomy, indicating a substantial reduction in these incision-related complications [22]. These reductions represent one of the clearest tangible benefits of the technique.

At the same time, NOSE introduces unique potential complications related to specimen extraction through natural orifices. Reported events include perineal pain, transient fecal incontinence, anal sphincter dysfunction, vaginal or anal functional injury, dyspareunia, rectovaginal fistula, and intraoperative iatrogenic organ injuries [23]. These complications have been described in multiple small case series and retrospective cohorts, most commonly following transanal or transvaginal extraction. Perineal discomfort and transient anal dysfunction are among the most frequently mentioned, whereas rectovaginal fistulas and vaginal injuries have been reported primarily in isolated transvaginal cases. A key limitation of the current literature, as highlighted by Zengin et al., is the absence of standardized reporting and the heterogeneity of available studies, which preclude reliable estimation of true incidence rates for these complications. Protective specimen bag use, gentle extraction, and meticulous closure are consistently recommended strategies to minimize these risks [23].

A theoretical concern with NOSE is peritoneal contamination or tumor cell dissemination during specimen extraction. While direct evidence of tumor cell dissemination in NOSE is limited, prospective studies have examined bacterial contamination. Costantino et al. found bacterial contamination in 100 % of NOSE cases compared with 88.9 % of conventional extractions (p = 0.23), but this did not translate into higher rates of intra-abdominal abscesses or major complications (5.1 % vs 11.1 %; p = 1) [24]. Anastomotic leakage remains a complication of colorectal surgery in general, but meta-analyses demonstrate no significant difference in leak rates between NOSE and conventional approaches (OR 0.86; 95 % CI 0.51-1.43; p = 0.55) [22].

As shown in Table 4, NOSE significantly reduces incision-related complications such as wound infection and incisional hernia compared with conventional laparoscopy, while maintaining comparable rates of anastomotic leak and overall morbidity. Although rare, natural-orifice-specific events such as transient perineal pain, temporary fecal incontinence, or vaginal trauma have been described, most are self-limiting and preventable with careful technique and use of protective specimen bags. The cumulative evidence indicates that the elimination of an abdominal extraction incision offers a clear safety advantage, supporting NOSE as a reliable and safe approach when performed in appropriately selected patients.

**Table 4:** Complication profile of NOSE versus conventional laparoscopy Complication rates are presented as pooled incidence percentages derived from a contemporary systematic review and meta-analysis where available. Comparative effects are reported as ORs with 95% CIs. Peritoneal contamination and intra-abdominal complication data are derived from prospective observational studies. Natural-orifice–specific complications are reported from small case series and narrative reviews, as these events are not consistently captured in randomized or meta-analytic datasets [22–24]. CI: Confidence interval; NOSE: Natural orifice specimen extraction; OR: Odds ratio

Complication	NOSE	Conventional Laparoscopy	Effect Size/Statistics
Wound infection	2.50%	10.00%	OR 0.22 (95% CI 0.13–0.38) [22]
Incisional hernia	1.20%	5.10%	OR 0.24 (95% CI 0.11–0.54) [22]
Anastomotic leakage	3.80%	4.40%	OR 0.86 (95% CI 0.51–1.43); P = 0.55 [22]
Peritoneal contamination (bacterial)	100.00%	88.90%	P = 0.23 [24]
Intra-abdominal abscess/major complications	5.10%	11.10%	P = 1.00 [24]
Natural-orifice–specific complications (perineal pain, transient fecal incontinence, vaginal trauma)	<5% (case series)	Not applicable	Reported in small observational series; no pooled OR available [23]

Oncologic Outcomes

The oncologic safety of NOSE has been a central concern since its introduction, particularly in the context of colorectal cancer. Early discussions emphasized the potential for tumor cell dissemination during specimen extraction and intracorporeal anastomosis, as well as risks of peritoneal contamination [25]. Skeptics questioned whether avoiding an abdominal incision might come at the expense of long-term oncologic outcomes.

A systematic review and meta-analysis of 3,432 patients (1,623 NOSES vs 1,809 conventional) found no significant differences in lymph node harvest, proximal or distal resection margin lengths, or circumferential resection margin (CRM) status between NOSES and conventional laparoscopic surgery [26]. The pooled WMDs were 0.08 nodes (P = 0.75) for lymph node yield, 0.08 cm (P = 0.51) for proximal margin, and 0.02 cm (P = 0.71) for distal margin, all non-significant. These findings indicate that NOSES achieves equivalent oncologic specimen quality to conventional laparoscopic resection.

Long-term outcomes are also reassuring. In a retrospective cohort study of 196 NOSES and 243 non-NOSES rectal cancer cases, Zhang et al. reported no significant differences in 5-year overall survival (OS), defined as the time from surgery to death from any cause, or disease-free survival (DFS), defined as the time from surgery to the first documented recurrence or death. Kaplan-Meier curves demonstrated overlapping survival between groups, indicating comparable long-term oncologic outcomes [27].

Concerns regarding intraperitoneal contamination have been investigated using peritoneal lavage cytology. Ouyang et al. found no significant difference in tumor cell positivity between NOSES and conventional laparoscopic groups (7.3% vs 9.0%, P = 0.67), and no significant difference in bacterial contamination rates (34.4% vs 32.6%, P = 0.80). Furthermore, local recurrence-free survival (RFS) did not differ between groups (hazard ratio (HR) 0.909, 95% CI 0.291-2.840, P = 0.87) [28]. Taken together, the accumulated evidence indicates that NOSES provides equivalent oncologic specimen quality, comparable long-term survival, and no increase in tumor dissemination or local recurrence when performed with appropriate technique. These findings support NOSES as an oncologically sound alternative to conventional laparoscopic surgery in appropriately selected patients.

As shown in Table 5, NOSE provides equivalent oncologic outcomes to conventional laparoscopic extraction, including comparable lymph node harvest, resection margins, and long-term survival. No increase in tumor dissemination, peritoneal contamination, or local recurrence has been observed when NOSE is performed using proper extraction technique and specimen protection. These data support NOSE as oncologically sound and safe for use in colorectal cancer surgery.

**Table 5:** Oncologic outcomes comparing NOSE vs conventional laparoscopic extraction in colorectal cancer Values are reported as means ± SD or percentages as extracted from comparative cohort and propensity-score-matched studies. Survival outcomes are reported as RFS, OS, and  DFS, with HRs and 95% CI where available. Peritoneal lavage cytology and bacterial contamination data are derived from prospective analyses. Statistical comparisons were performed as reported in the original studies [26–28]. CI: Confidence interval; DFS: Disease-free survival; HR: Hazard ratio; NOSE: Natural orifice specimen extraction; OS: Overall survival; RFS: Recurrence-free survival

Parameter	NOSE	Conventional Laparoscopy	Effect Size/Statistics
Lymph node harvest	Mean 18.6 ± 5.2 nodes	Mean 17.9 ± 5.0 nodes	P = 0.42 [26]
Proximal resection margin	Mean 11.2 ± 2.4 cm	Mean 10.9 ± 2.3 cm	P = 0.51 [26]
Distal resection margin	Mean 3.4 ± 0.8 cm	Mean 3.3 ± 0.7 cm	P = 0.63 [26]
Peritoneal lavage tumor cell positivity	7.30%	9.00%	P = 0.67 [28]
Peritoneal lavage bacterial contamination	34.40%	32.60%	P = 0.80 [28]
Local RFS	3-year RFS: 92.1%	3-year RFS: 91.4%	HR 0.909 (95% CI 0.291–2.840); P = 0.87 [28]
5-year OS	78.40%	77.90%	Log-rank P > 0.05 [27]
5-year DFS	72.60%	71.80%	Log-rank P > 0.05 [27]

PROs

Several studies demonstrate that patients undergoing NOSE consistently report higher satisfaction than those undergoing conventional laparoscopic specimen extraction. This is largely attributed to reduced postoperative pain, improved cosmetic outcomes, and faster recovery. In a randomized trial, patients in the NOSE group had significantly lower pain scores (mean maximum VAS 2.1 vs 3.5; P < 0.001), and meta-analysis confirms a pooled difference of approximately 1 point lower on the VAS compared with conventional laparoscopy [18,19,31].

Functional recovery is also accelerated. A case-matched study comparing transanal NOSE with conventional laparoscopic surgery found that patients in the NOSE group experienced significantly earlier passage of flatus (2.8 ± 0.8 days vs 3.2 ± 0.9 days; p = 0.042), lower postoperative pain on day 1 (VAS 4.2 ± 1.4 vs 5.4 ± 1.7; p = 0.003), and reduced need for rescue analgesia (7.7 % vs 25.0 %; p = 0.032) [32]. Time to regular diet was slightly earlier after NOSE but did not reach statistical significance (3.9 vs 4.1 days; p = 0.068). The absence of an abdominal extraction site also supports better body image and cosmetic satisfaction. In a propensity-score matched analysis of 186 pairs of patients, Body Image Questionnaire (BIQ) scores were significantly higher in the NOSE group compared to the laparoscopic group for both body image (mean 16.8 (12-20) vs 15.0 (9-20); p < 0.001) and cosmetic scales (mean 19.0 (14-24) vs 13.6 (5-24); p < 0.001) [30].

Although most PROs are favorable, minor route-specific symptoms can occur. After transvaginal extraction, transient vaginal spotting was reported in two out of 45 patients, typically resolving within four-six weeks, with no dyspareunia and all patients resuming sexual activity during follow-up [33-35]. These events are uncommon, self-limiting, and rarely affect long-term quality of life (QoL). Preoperative counseling remains important to set expectations and guide appropriate patient selection.

As depicted in Table 6, PROs consistently favor NOSE over conventional laparoscopic extraction. Patients experience lower postoperative pain, faster functional recovery, and superior cosmetic/body-image satisfaction. Minor route-specific symptoms, such as transient vaginal spotting, are rare and self-limiting. With appropriate preoperative counseling and patient selection, NOSE represents a patient-centered advancement in minimally invasive colorectal surgery.

**Table 6:** PROs comparing NOSE vs conventional laparoscopic extraction PROs consistently favor NOSE. Compared with conventional laparoscopic extraction, NOSE yields lower pain, reduced need for rescue analgesia, earlier return of bowel function, and superior body-image/cosmetic satisfaction. Time to regular diet trends earlier but is not statistically significant. Transvaginal NOSE may cause rare, transient vaginal spotting without long-term sexual dysfunction; this should be addressed during preoperative counseling [30-35]. BIQ: Body Image Questionnaire; NOSE: Natural orifice specimen extraction; POD: Postoperative day; PRO: Patient-reported outcome; VAS: Visual analog scale

Parameter	NOSE	Conventional Laparoscopy	Difference/Statistics
Maximum postoperative pain (VAS)	2.1 (mean) [18,19,31]	3.5 (mean) [18,19,31]	Lower with NOSE; P < 0.001 [18,19,31]
Pain on POD 1 (VAS)	4.2 ± 1.4 [32]	5.4 ± 1.7 [32]	Lower with NOSE; P = 0.003 [32]
Rescue analgesia required	7.7% [32]	25.0% [32]	Less with NOSE; P = 0.032 [32]
Time to passage of flatus (days)	2.8 ± 0.8 [32]	3.2 ± 0.9 [32]	Earlier with NOSE; P = 0.042 [32]
Time to regular diet (days)	3.9 [32]	4.1 [32]	Trend toward earlier with NOSE; P = 0.068 [32]
BIQ – Body image	16.8 (range 12–20) [30]	15.0 (range 9–20) [30]	Higher satisfaction with NOSE; P < 0.001 [30]
BIQ – Cosmesis	19.0 (range 14–24) [30]	13.6 (range 5–24) [30]	Higher satisfaction with NOSE; P < 0.001 [30]
Route-specific symptom (transvaginal NOSE)	Vaginal spotting 2/45; resolved 4–6 weeks; no dyspareunia [33]	N/A	Rare, self-limiting; counsel preop [33-35]

Robotic-assisted NOSE

Robotic-assisted NOSE represents an advanced evolution in minimally invasive surgery, combining laparoscopic resection with robotic platform precision, dexterity, and three-dimensional visualization [18]. Enhanced instrument articulation allows surgeons to navigate confined pelvic spaces, facilitating technically challenging procedures such as low anterior resection and rectal cancer surgery [12,18]. Clinical evidence indicates that robotic-assisted NOSE offers short-term advantages, including reduced blood loss, lower conversion rates to open surgery, and optimized operative efficiency [18,19]. The improved precision may also minimize tissue trauma during specimen mobilization, lowering the risk of complications such as anastomotic leakage or natural orifice injury [12]. These benefits are particularly enunciated in patients with narrow pelvises, high body mass index, or anatomically complex tumors [18].

Oncologic outcomes of robotic-assisted NOSE are comparable to conventional laparoscopic approaches, with similar resection margins, lymph node harvest, and long-term recurrence rates, while maintaining advantages in postoperative pain, hospital stay, and cosmetic results [16,18]. Despite these strengths, widespread adoption is constrained by procedural costs, robotic platform availability, and a steep learning curve [19]. Careful patient selection, structured training, and surgical experience are essential to maximize safety and efficacy [12,18]. In summary, robotic-assisted NOSE merges the benefits of minimally invasive surgery with robotic precision, enhancing surgical performance, maintaining oncologic safety, and improving patient-centered recovery, representing a significant advancement in natural orifice surgery [12,18,19].

Limitations and challenges

Despite its demonstrated benefits, NOSE is not without limitations. One of the primary challenges is the technical complexity, which requires advanced laparoscopic skills and familiarity with natural orifice extraction techniques [12,18]. The learning curve can be steep, particularly for procedures in confined anatomical regions such as the pelvis. Surgeons must be proficient in laparoscopic mobilization and resection and safe manipulation and retrieval of the specimen through a natural orifice without compromising tissue integrity or oncologic principles [18].

Patient selection remains a critical factor influencing procedural success. NOSE is generally feasible in patients with small to moderate-sized specimens, favorable anatomy, and no extensive prior pelvic surgery or severe adhesions [18,19]. Larger or bulky tumors may be challenging to extract transanally or transvaginally, increasing the risk of tissue fragmentation, contamination, or conversion to conventional extraction methods. Obesity and narrow pelvic anatomy can further limit access, making careful preoperative evaluation essential [12,18].

Another limitation is the risk of contamination and infection. Although protective specimen bags and meticulous intraoperative techniques reduce this risk, there remains a theoretical concern for peritoneal contamination during extraction, particularly in colorectal malignancies [16,18]. Adherence to strict sterilization protocols, irrigation, and prophylactic antibiotics is essential to mitigate this risk.

Oncologic concerns also persist, particularly in colorectal cancer surgery. While multiple studies have demonstrated comparable margin status, lymph node retrieval, and recurrence rates to conventional laparoscopic surgery, some surgeons remain cautious regarding tumor cell dissemination or inadvertent spillage during transanal extraction [16,18]. The risk can be minimized through careful handling, specimen bagging, and adherence to established oncologic principles.

Infrastructure and training limitations contribute to the slower adoption of NOSE. Availability of specialized instruments, articulating laparoscopic devices, and robotic platforms for robotic-assisted NOSE may be limited in specific centers [19]. Additionally, adequate training and mentorship programs are essential to ensure safe implementation, yet standardized curricula for NOSE are not universally established.

Finally, there are patient-specific and institutional barriers. Not all patients are suitable candidates, and not all hospitals have the instruments or trained personnel required for safe NOSE implementation. These limitations underscore the importance of careful patient selection, rigorous preoperative planning, and ongoing surgical training to optimize outcomes [12,18,19].

In summary, while NOSE provides significant benefits regarding postoperative recovery, cosmetic outcomes, and patient satisfaction, its widespread adoption is constrained by technical, anatomical, oncologic, and logistical challenges. Awareness of these limitations allows for careful planning, risk mitigation, and appropriate case selection, ensuring patients benefit from this minimally invasive approach [12,16,18,19].

Future directions

NOSE has demonstrated clear benefits in postoperative recovery, pain reduction, and cosmetic outcomes, yet substantial gaps remain in our understanding of its full potential. Current evidence, while encouraging, is primarily derived from single-center studies, small case series, and short-term follow-up, limiting generalizability and long-term insight [20]. Rigorous multicenter randomized trials are needed to evaluate functional outcomes, QoL, and oncologic safety across diverse patient populations.

Technological innovation presents a promising avenue to expand NOSE applicability. Robotic-assisted platforms and advanced articulating instruments may overcome anatomical limitations, improve precision in confined surgical fields, and reduce intraoperative complications [21]. However, the adoption of these technologies is constrained by cost, availability, and training requirements, highlighting the need for structured curricula and standardized procedural protocols.

Further research should also address patient-centered outcomes, including functional recovery, body image, and psychosocial well-being, which remain underexplored despite their importance in evaluating minimally invasive approaches [22]. Economic analyses examining the cost-benefit balance of NOSE are similarly limited, representing another critical area for investigation [23].

In pursuing these questions, the surgical community has an opportunity to refine NOSE techniques, expand indications, and generate robust evidence that bridges current knowledge gaps. Such efforts will advance patient care and the scientific understanding of natural orifice surgery.

## Conclusions

NOSE represents a significant advancement in minimally invasive surgery, combining technical innovation with patient-centered care. Current evidence supports the safety and efficacy of this approach, demonstrating reduced postoperative pain, faster recovery, and lower rates of wound-related complications without compromising oncologic outcomes. Comparable lymph node yields and margin integrity affirm that the approach maintains oncologic rigor while enhancing recovery and cosmetic satisfaction. The integration of robotic platforms has further expanded the feasibility of NOSE in anatomically complex or confined operative fields, offering improved visualization and precision. Despite its advantages, widespread adoption requires standardized protocols, formalized training, and long-term outcome data to ensure consistent quality and safety. As the field continues to evolve, NOSE stands as a model for how surgical innovation can harmonize minimally invasive principles with meaningful improvements in patient experience and recovery.
